# Ventilation-induced jet suggests biotrauma in reconstructed airways of the intubated neonate

**DOI:** 10.1098/rsif.2019.0516

**Published:** 2020-01-08

**Authors:** Eliram Nof, Metar Heller-Algazi, Filippo Coletti, Dan Waisman, Josué Sznitman

**Affiliations:** 1 Department of Biomedical Engineering, Technion–Israel Institute of Technology, Haifa 3200003, Israel; 2 Faculty of Medicine, Technion–Israel Institute of Technology, Haifa 3200003, Israel; 3 Department of Aerospace Engineering and Mechanics, University of Minnesota, Minneapolis, MN 55455, USA; 4 Department of Neonatology, Carmel Medical Center, Haifa 3436212, Israel

**Keywords:** neonates, lung injury, biological fluid mechanics, biotrauma, respiratory distress syndrome

## Abstract

We investigate respiratory flow phenomena in a reconstructed upper airway model of an intubated neonate undergoing invasive mechanical ventilation, spanning conventional to high-frequency ventilation (HFV) modes. Using high-speed tomographic particle image velocimetry, we resolve transient, three-dimensional flow fields and observe a persistent jet flow exiting the endotracheal tube whose strength is directly modulated according to the ventilation protocol. We identify this synthetic jet as the dominating signature of convective flow under intubated ventilation. Concurrently, our *in silico* wall shear stress analysis reveals a hitherto overlooked source of ventilator-induced lung injury as a result of jet impingement on the tracheal carina, suggesting damage to the bronchial epithelium; this type of injury is known as biotrauma. We find HFV advantageous in mitigating the intensity of such impingement, which may contribute to its role as a lung protective method. Our findings may encourage the adoption of less invasive ventilation procedures currently used in neonatal intensive care units.

## 1. Introduction

Preterm birth complications are the leading cause of newborn infant mortality [1]. More than one in every 10 global births are premature, a rate that is widely expected to increase in years to come [2,3]. Infant respiratory distress syndrome (IRDS), a disorder in which surfactant deficiency impairs breathing, represents the primary cause among premature deaths [4]. The two most effective methods for treating IRDS in neonates are surfactant replacement therapy and mechanical ventilation, often employed concurrently [5]. Unfortunately, conventional mechanical ventilation (CMV) is known to cause ventilator-induced lung injury (VILI), wherein structural changes to the airway tissue, such as volu- and barotrauma, as well as cellular injury, known as biotrauma [6], can lead to systemic dysfunction and ultimately death. Seminal *in vivo* investigations nearly 40 years ago [7,8] led to the development of an alternative ventilation strategy known as high-frequency ventilation (HFV). This method supplies small tidal volumes of air at high respiratory breathing rates to curtail VILI effects while guaranteeing carbon dioxide removal. The initial optimism among the clinical community [9,10] that HFV would become a general remedy for neonatal respiratory insufficiency has been mitigated by more than 30 years of data that come short in delivering conclusive evidence to its purported advantages [11,12]. Still, HFV is employed in neonatal intensive care units worldwide both as a rescue therapy and in standard care [11,13]. Yet, the ongoing lack of consensus in clinical guidelines results in a trial-and-error approach for initializing and adjusting ventilator settings [14]. Furthermore, the disparity between HFV protocols hinders efforts to cohesively integrate the data on these young patient populations [12], whose enrolment in clinical studies remains sensitive because of ethical considerations [15,16].

Past *in vivo* studies have supported the notion of improved oxygenation and a reduction in lung pathology severity in premature animals treated with HFV [17–21]. Theoretical models on the mechanistic foundations of this counter-intuitive ventilation approach, namely oscillating tidal volumes smaller than the anatomical dead space, have advanced a number of mechanisms collectively responsible for the enhanced gas exchange counterbalancing the reduction in tidal volume [22,23]. These include direct alveolar ventilation, inter-regional asynchronous mixing (pendelluft), steady streaming following asymmetry between flow profiles during inhalation and exhalation, mixing due to secondary flows and molecular diffusion in the deep acinar regions. Though the rationale behind these flow mechanisms is acknowledged, few if any experimental studies have directly observed them. Moreover, *in vivo* flow data in neonatal HFV are still beyond reach despite progress with magnetic resonance imaging-based modalities using hyper-polarized gases [24,25]. A recent numerical simulation on a premature neonatal lung model [26] recognized the existence of various transport mechanisms, but the debate persists on whether flows under HFV are indeed fundamentally different from CMV [27], as is often described [28,29]. Several studies have alluded to the increase in convective-driven recirculation from local secondary flows [30,31] and pendelluft [32–34] near reversal times between inhalation and exhalation. However, these phenomena are known to be transient relative to the breathing cycle [35], leaving their overall contribution to HFV-specific gas mixing unknown. Han & Hirahara [36] attributed enhanced mixing to steady streaming measured in a symmetric double bifurcation, while others have reported a minimal [37], if not negative [38], correlation between steady streaming and ventilation frequency. Following a dearth of evidence, the prospect of establishing clinical HFV guidelines calls for a better mechanistic understanding of the specific transport mechanisms that govern this ventilation mode. Such quantification is seen as a crucial step towards improving HFV safety and efficacy [39,40].

To shed new light on this ongoing question, we present *in vitro* experiments quantifying the convective flow characteristics of HFV in a full-size, reconstructed neonatal upper airway model under intubation. We employ high-speed tomographic particle image velocimetry (tomoPIV) to resolve transient, three-dimensional flow fields and screen a physiological spectrum of frequency and tidal volumes pertinent to HFV. In particular, the intubation tube, a common feature of clinical HFV protocols, has often been absent or overlooked in previous flow transport investigations [30,38,41]. In its presence, we observe the systematic formation of a high-momentum region analogous to a synthetic jet and quantify its temporal evolution as a function of ventilation frequency. This transport feature, present in both CMV and HFV, carries the most dominant flow feature under intubation. Such results suggest that intubation-related ventilation effects may overshadow other convective transport mechanisms in upper airways. Furthermore, our *in silico* analysis pinpoints a hitherto unrecognized source of injury to the bronchial epithelium due to high wall shear stresses (WSSs) at the carina walls following jet impingement—a phenomenon consistent with lung *biotrauma* [42]. To the best of our knowledge, we present what may be a new paradigm on convective flows in upper airways of the intubated neonate.

## 2. Results and discussion

### 2.1. Convective transport in upper airways

**Figure 1.**
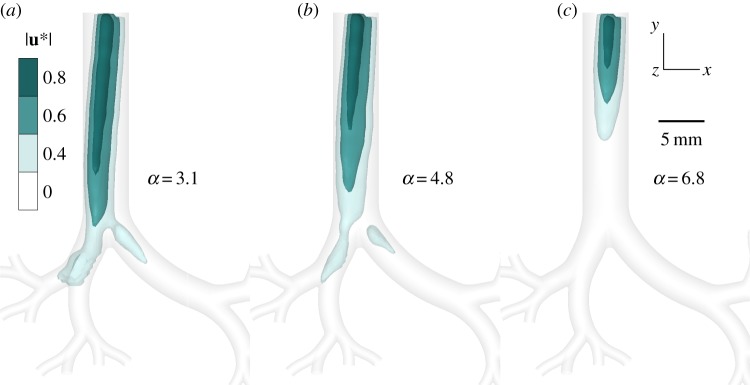
Convective flows in reconstructed upper airways of an intubated preterm neonate undergoing invasive ventilation. Three-dimensional velocity vector fields are experimentally extracted via tomoPIV and averaged over 30 phase-locked ventilation cycles. Elongated, jet-like regions of high momentum introduced by the endotracheal tube (ETT) are visualized by plotting iso-surfaces of the normalized velocity magnitude $\left|u^{*}\right|$. The jets are depicted according to three levels of iso-surfaces: a high-velocity core (80% of maximum velocity) sheathed by partially transparent lower velocities, i.e. 60% and 40%, respectively. Three ventilation modes spanning clinically relevant values of HFV (table 1) are evaluated at the instance of peak jet strength: *α* = 3.1 at *t*/*T* = 0.21 (*a*), *α* = 4.8 at *t*/*T* = 0.27 (*b*) and *α* = 6.8 at *t*/*T* = 0.34 (*c*), where *α* is the dimensionless frequency (Womersley number). The size and strength of the jet core reduces as *α* increases and correspondingly tidal volume decreases (i.e. *Q* is fixed). The evolution of these jets over the entire inhalation phase is provided in electronic supplementary material, movie S1 for the three cases. (Online version in colour.)

Time-resolved flow measurements are obtained in a three-dimensional printed model of upper airways, corresponding to an intubated preterm neonate (see Methods). Specifically, velocity vector fields are extracted from high-speed tomoPIV over repeating (*N* = 30) phase-locked ventilation cycles conducted in a glycerol/water solution following dynamic similarity (see Methods). In this region of the lungs, where inertial effects dominate over diffusion (Pe ≫ 1; see governing equations in Methods), ventilation is predominantly convective. Flow exiting the intubation tube into the trachea during inhalation develops into an elongated region of high momentum, a configuration consistent with a well-known phenomenon of a jet discharging from an orifice into a larger space [43]. We visualize this jet at peak strength by plotting velocity magnitude isosurfaces (figure 1) as a function of the dimensionless frequency, i.e. Womersley number *α*. The Womersley number measures the relative importance of unsteadiness (i.e. inertial acceleration) due to changes in ventilation frequency (see experimental set-up in Methods for correspondence with clinical ventilation settings). The complete temporal evolution of the three jets over a full inhalation phase is presented in the electronic supplementary material, movie S1. For the lowest frequency (*α* = 3.1), the jet impacts the first bifurcation and separates into the two main bronchi, whereas for the highest frequency (*α* = 6.8) the jet core dissipates upstream within the tracheal entrance. We observe slight deviations of the jet towards the right bronchus as a result of the asymmetric pressure differences between the right and left lung (‘left’ and ‘right’ refer to the anatomical orientation of the lungs). This pressure difference can be understood by considering the elongation of the left bronchus first generation relative to the right, as depicted in figure 1, which owing to equal pressure conditions at the outlets of the experiment accounts for a relative flow resistance gradient favouring flow to the right bronchus. While the pressure outlet boundary conditions of the *in vitro* set-up are not strictly physiological (see Methods), whereas *in vivo* local ventilation distribution patterns are the outcomes of local pressure gradients applied distally across specific lung lobes, the presence and strength of the ventilation-induced jet upstream in the trachea would not be significantly affected.

**Figure 2.**
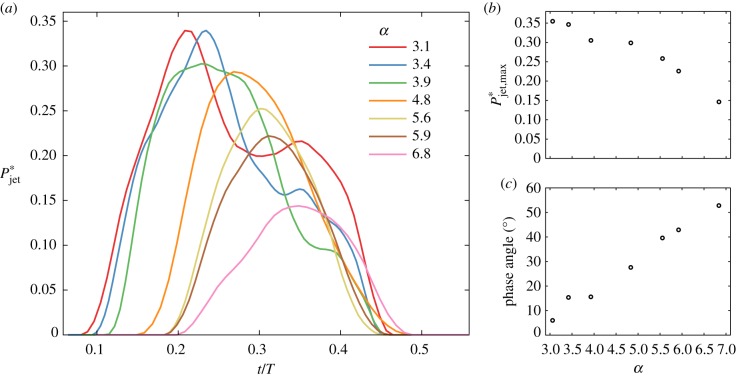
Momentum analysis of the intubation jet from *in vitro* three-dimensional flow measurements. (*a*) Evolution of the dimensionless momentum $P_{\mathrm{jet}}^{*}$ over a characteristic inhalation (i.e half cycle). Here $P_{\mathrm{jet}}^{*}$ is based on a 50% velocity magnitude threshold relative to the maximum velocity |u*|_max_ and then normalized by the total momentum across the entire upper airway model. The jet’s strength decreases with increasing ventilation frequency. (*b*) The extracted peak magnitude of the jet momentum $P_{\mathrm{jet},\max}^{*}$ is shown as a function of dimensionless frequency. (*c*) The growing influence of flow unsteadiness and asynchrony (relative to invasive ventilation) with increasing frequency is underlined in the phase angles of $P_{\mathrm{jet},\max}^{*}$ (i.e. phase lag). (Online version in colour.)

We next quantify the jet’s structural changes for the parameter space of table 1, reported in values equivalent to air. Here, we choose an integral approach [44] for measuring the momentum enclosed within the jet, i.e. $P_{\mathrm{jet}}=\int \rho |u|\;\text{d}V$, where |u| is the velocity magnitude and *ρ* is the fluid density. The volume integral ∫dV extends over the subdomain of the jet determined by a velocity magnitude threshold greater than or equal to 50% of the maximum velocity (|**u**|_max_) at any given phase in the full model. We quantify the jet momentum, $P_{\mathrm{jet}}^{*}$, over the inhalation phase as a function of *α* (figure 2*a*), where the asterisk (*) denotes normalized values relative to the instantaneous total momentum in the full imaged lung domain. At its peak strength, the jet at low *α* captures approximately a third of the total energy while curves at larger values of *α* reach maxima of less than or equal to 15%. Plotting peak values of the jet momentum curves ($P_{\mathrm{jet},\max}^{*}$) underlines a monotonically decreasing trend (figure 2*b*) in line with the qualitative observations of figure 1. Note that altering the velocity magnitude threshold |**u**|_max_ in our analysis does not change the outcome. Further extraction of phase angles of $P_{\mathrm{jet},\max}^{*}$ reveals a phase lag that increases almost linearly with *α* (figure 2*c*), indicating the growing influence of flow unsteadiness with higher operative frequencies. This phenomenon leads to increasing asynchrony between invasive ventilation and ensuing respiratory flows in the upper airways. We return to the implications of this jet phenomenon (i.e. strength and phase lag) in relation to WSS and biotrauma below.

**Table 1.** Experimental flow parameters spanning clinically relevant modes of HFV. Ventilation frequency is given both in dimensionless form (i.e. Womersley number, *α*) and the equivalent ventilation frequency in air, *f*, calculated via dynamic similarity. Re_0_ denotes the mean flow Reynolds number in the trachea (also referred to as airway generation 0), which is kept constant via the ventilation efficiency parameter *f* × *V*_*T*_ (frequency × tidal volume).

*f* (Hz)	*V*_*T*_ (ml)	*α*	Re_0_
3.8	2.5	3.1	300
4.8	2.0	3.4	300
6.3	1.5	3.9	300
9.5	1.0	4.8	300
12.7	0.75	5.6	300
14.3	0.67	5.9	300
19.0	0.5	6.8	300

We note that, under the ventilation conditions examined, temporal flow fluctuations or other localized instabilities characteristic of turbulence are absent, including at peak inhalation when flow rates and hence the mean Reynolds number are maximal (Re_0_ < 500). The Reynolds number measures the importance of inertial effects relative to viscous forces and can help predict the onset of turbulence. Our results are in line with experimental investigations on bifurcating tubes reporting critical Reynolds numbers [45] between 680 and 1300. While turbulent structures may arise in adult upper airways, particularly downstream of the glottal constriction [46,47], flow patterns under physiological breathing conditions relevant to the premature neonate are well within a laminar regime. This stands in contrast to mentions of turbulence as a ventilation-enhancing mechanism in adult HFV [28]. Considering that our reconstructed geometry is based on dimensions of a relatively large (approx. 2 kg) preterm neonate, we anticipate that the assumption of laminar flow extends more broadly to the general preterm population.

**Figure 3.**
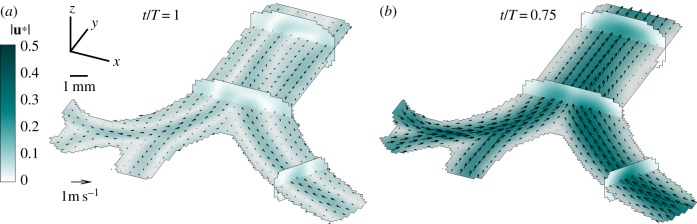
Experimental measurements of flow patterns during the exhalation phase under HFV. TomoPIV data shown at *α* = 4.8 are averaged over 30 phase-locked cycles and presented at two distinct instants: (*a*) the end of exhalation (*t*/*T* = 1) and (*b*) peak exhalation (*t*/*T* = 0.75). Four span-wise cross-sections across the tree of the resulting flow field capture the main three-dimensional flow features. (Online version in colour.)

In parallel, an often cited phenomenon thought to enhance gas mixing in HFV is the so-called pendelluft effect, whereby neighbouring regions in the lung fill and empty asynchronously, causing inter-regional flow exchange [48,49]. Yet, the occurrence of pendelluft is expected only near flow reversal times (as shown in figure 3*a*), i.e. between inhalation and exhalation, and quantitative evidence supporting the significance of this phenomenon is still lacking. Experimentally, we observe such a pendelluft effect at the highest frequency (*α* = 6.8) during the short transition (less than 3% of the cycle) from inhalation to exhalation (*t*/*T* = 0.4), when velocities approach zero (see electronic supplementary material, figure S3). Similarly, Bauer *et al.* [35] could not identify pendelluft in their neonatal upper airway model, which they reasoned may have been attributed to insufficient temporal resolution for capturing the precise instance of flow reversal. Despite the relatively high frame rate provided by our high-speed camera set-up (1000 frames per second (fps)), additional evidence of pendelluft is not observed in our reconstructed airway models and may be a feature limited to deeper lung regions.

On the other hand, the exhalation phase during breathing (figure 3) exhibits markedly different characteristics from those observed during inhalation. Converging flows entering from daughter branches dictate the dominant flow pattern of jet-like structures much weaker than those during inhalation that merge right above each bifurcation, as seen most clearly during peak exhalation (figure 3*b*). This flow topology is familiar to studies in both neonatal and adult airways [35,50]. Here, we note only a minor increase in mean velocity with rising *α* (see electronic supplementary material, figure S3), matching recent findings [38] where the expiratory phase remains largely consistent for frequencies varying from CMV to HFV.

Lateral mixing by secondary flows can play an important role in mass transport inside curved tubes via enhancement of the effective diffusivity of the solute [51]. Kamm [52] suggested that secondary flows may promote augmented axial dispersion at higher oscillatory frequencies such as in HFV. Spanning the parameter space with constant values of *f* × *V*_*T*_ is based on a linear relationship with ventilation efficiency [53], though other studies [20,21,54] have suggested varying the exponents *a* and *b* in $f^{a}\times V_{T}^{b}$, which may modify these results. The Dean number, Dn=ReD/2r, where *r* denotes the radius of curvature of an airway of diameter *D*, measures the strength of the secondary flow features relative to the primary axial flow. We recall that Re is held constant in our investigation (and so is the product *f* × *V*_*T*_), such that the strength of Dean-related secondary flows (Dn ∼ Re) remains largely insensitive to changes in *α*; this is in agreement with Bauer *et al.* [31], who used a constant ventilation efficiency. Altogether, neither we nor past studies find substantive evidence of the significance of the aforementioned transport mechanisms (i.e. turbulence, pendelluft and secondary flow mixing). Instead, our experiments underscore the inhalation jet as the single, dominant convective flow signature for intubated ventilation in the upper airways of the neonate.

### 2.2. Wall shear stress and biotrauma

The direct consequences of a jet flow in upper airways have to date not been widely appreciated in the clinical literature. A study from almost 30 years ago found increased WSS in a neonatal tracheal model as a result of an impinging jet exiting an off-centred cuffed intubation tube, concluding that efforts should be made by clinicians to centre the tube and thereby possibly reduce trauma in the patient [55]. No subsequent work directly discussed this topic, perhaps owing to the conventional assumption that tracheal damage is primarily inflicted by the pressure exerted on the wall by the inflated cuff of the intubation tube [56]. Recent insights into the cellular response to repeated shear stress have indicated that there are more complex contributors to ventilator-related injury than previously thought. Although studies on the pathogenesis of VILI have historically focused on biophysical injury due to mechanical damage, such as lung over-distention and the collapse of alveoli, more recent works reveal a subtler form of injury, termed biotrauma [6,42]. This concept predicts that the stresses inflicted by mechanical ventilation at the cellular level induce the release of mediators that trigger or worsen lung injury, possibly leading to systemic organ failure. Gattinoni *et al.* [57] outlined the cascade in which mechanical strain activates macrophages and epithelial cells to produce interleukin-8, a cytokine that recruits neutrophils causing inflammation. Commenting on a recent clinical meta-analysis on the link between driving pressure and postoperative pulmonary complications [58], Jamaati *et al.* [59] proposed that airflow-related shear stress may play a larger role in contributing to VILI than previously thought.

**Figure 4.**
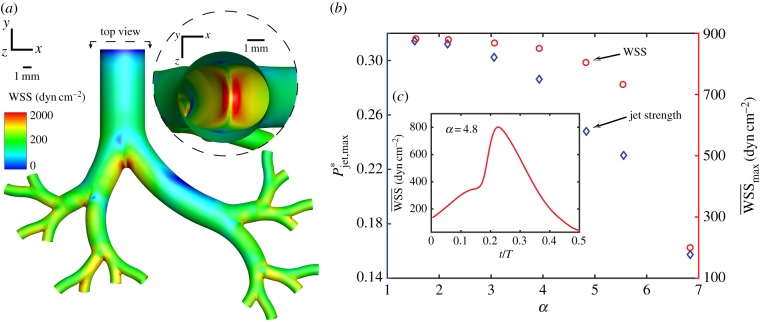
Numerical WSS analysis in the reconstructed neonatal upper airway due to an impinging jet arising from the endotracheal intubation tube during mechanical ventilation. (*a*) Airway WSS magnitude contours are displayed at peak momentum jet strength (*α* = 4.8 at *t*/*T* = 0.27) with a top view (see inset) into the trachea revealing a concentrated area of high WSS at the carina (first bifurcation). Mean WSS ($\bar{\text{WSS}}$) is calculated over the area of this bifurcating zone for each ventilation case spanning CMV and HFV. (*b*) $\bar{\text{WSS}}$ (circles) at peak momentum, $P_{\mathrm{jet},\max}^{*}$ (diamonds), as a function of *α*. We observe two similar, monotonically decreasing trends indicating a reduction in ${\bar{\text{WSS}}}_{\max}$ attributed to higher ventilation frequency. (*c*) Temporal evolution of $\bar{\text{WSS}}$ for *α* = 4.8, with the peak value corresponding to ${\bar{\text{WSS}}}_{\max}$ plotted in (*b*). (Online version in colour.)

Following our measurements of the high-momentum jet region in the trachea (figure 1) combined with the observation that HFV protocols using lower tidal volumes reduce its strength (figure 2), we explore the relationship between the jet phenomenon and airway WSS. Because of the reliance of PIV on the statistical tracking of seeded particles (see tomoPIV processing steps in Methods), resolving near- and on-wall regions where particle density is low remains challenging. To access wall-related flow features, we turn to computational fluid dynamics (CFD) following validation against experimental tomoPIV data (see details in Methods). We repeat the integral momentum analysis spanning HFV to CMV (electronic supplementary material, table S1) and observe jet attenuation and phase lag trends (electronic supplementary material, figure S2) that fall in line qualitatively and quantitatively with our experimental results. We evaluate the hypothesis that the reduction in jet strength associated with higher *α* correlates with diminishing WSS. Figure 4*a* exhibits WSS on the airways during ventilation shown at *α* = 4.8. We note areas of high WSS concentrated near the bifurcations, with the first carina (see inset) proving the most susceptible, as it is impacted by the entire jet before it separates and attenuates into the next airway generation. In figure 4*b*, we plot the maximum jet momentum ($P_{\mathrm{jet},\max}^{*}$) values for each ventilation case, showing a declining trend corroborating our experimental measurements (figure 2*b*). Finally, we plot the mean WSS at the first bifurcation at peak jet momentum strength, denoted ${\bar{\text{WSS}}}_{\max}$, and observe a similar, monotonically decreasing trend with a reduction by an order of magnitude between CMV and the highest frequency of HFV. Additionally, in figure 4*c* we exemplify for the case of *α* = 4.8 the temporal evolution of the spatially averaged WSS values across the first bifurcation, noting that the profile is largely in phase with the transient evolution of $P_{\mathrm{jet}}^{*}$ shown earlier in figure 2*a*, with a peak at approximately *t*/*T* = 0.27. We note that the use of rigid walls during our numerical analysis reflects a non-compliant airway, a consequence of surfactant deficiency common in premature neonates [13]. A computational investigation into the effects of airway tissue flexibility reported an increase in WSS values at the bifurcations of an adult airway model undergoing slow breathing [60], indicating that our simulated values may be conservative.

We next investigate the physiological relevance of the extracted WSS values in relation to shear-induced lung injury. Although cell and tissue damage is closely related to the amplitude of cyclic stretch, the exact mechanisms leading to adverse outcomes in patients with or without lung injury remain unclear. A study that exposed rat alveolar epithelial cells to repeated deformations found that reducing the amplitude by superimposing small cyclic deformations on an equivalent base significantly reduced cell death compared with large-amplitude deformations with the same peak [61]. Sidhaye *et al.* [62] examined *in vitro* the effects of flow shear stress on human bronchial epithelial cells and in intact mice trachea that was subsequently excised and analysed *post vivo*. The authors found that values of WSS between 1 and 10 dyn cm^−2^ modulate paracellular permeability across the epithelial barrier, indicating a plausible link to VILI [63]. In the absence of *in vivo* data, Green [64] numerically studied cough-induced WSS in an adult airway model and postulated that the measured peak WSS values (10–200 dyn cm^−2^) could play an important role in the inflammatory process of respiratory disease. In our reconstructed neonatal model, local WSS values near the bifurcations reach 2 × 10^3^ dyn cm^−2^, exceeding critical values mentioned in the literature by at least an order of magnitude, indicative of the potential for significant cell and tissue damage. Furthermore, figure 4*b* shows that increased *α* reduces peak $\bar{\text{WSS}}$ to values beyond those generated at rest during breathing [62] (0.5–3 dyn cm^−2^), suggesting that all frequencies under intubated ventilation pose an elevated risk. Further experimental investigation is needed to verify that shear stress on airway walls leads to epithelial injury. Furthermore, a quantification of the detrimental effects must be assessed in order to weigh the impact relative to other injurious stimuli.

Considering the clinical variability in choosing an endotracheal tube insertion depth, a more distally placed tip would probably exacerbate these results. It should be emphasized that, during mechanical ventilation, the effects of shear stress are repeated at rates of the order of 10^6^ cycles/day. Some infants, especially those born extremely prematurely (i.e less than 28 weeks), are kept on ventilatory support for as long as several months, which probably amplifies the cumulative damage induced in the young patients. Studies have only recently examined the long-term effects of mechanical ventilation on preterm infants [65]. We raise the possibility that the jet arising from the intubation tube increases WSS on the bronchial epithelium to levels sufficiently high to cause cellular damage and thus biotrauma. In turn, HFV and in particular reducing tidal volume for the same flow rate (*f* × *V*_*T*_) may mitigate such adverse effects, thereby offering lung protective benefits relative to CMV. While our findings may offer support to claims for the benefit of HFV in reducing lung injury, within the scope of our present analysis our efforts have been limited to an upper airway model. As such, these are thus restricted in delivering direct insight into the limits of operating clinical ventilation protocols combining high frequency and low tidal volume towards ventilation efficiency (i.e. oxygenation and CO_2_ clearance), owing to a reduction in tidal volume. In ultimately seeking an optimum ventilation guideline, future studies are needed to better understand how the mechanistic flow effects in the upper airways influence gas exchange in the deeper respiratory regions of the lungs. Finally, though our analysis concentrates on dimensions and flow conditions relevant to the neonate, similar trends may exist in paediatric and adult populations.

## 3. Methods

**Figure 5.**
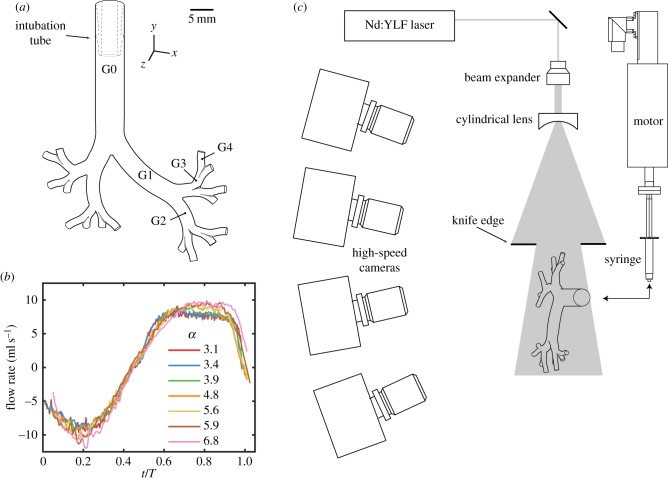
Schematic illustration of the experimental set-up. (*a*) The silicone airway model undergoes (*b*) sinusoidal oscillatory ventilation (experimental data shown as a function of dimensionless frequency *α*). (*c*) Four high-speed cameras, a high-energy laser and optics constitute the hardware components for tomoPIV used to measure the instantaneous, three-dimensional velocity fields. (Online version in colour.)

Our experimental and numerical investigations revolve around the use of a morphometrically faithful model of reconstructed neonatal upper airways. This airway model (figure 5*a*) is based on a previously published hybrid geometry [66], following the seminal works of Weibel [67] and Horsfield *et al.* [68]. The computer-aided design (CAD) model was homothetically scaled down to the size of an approximately 2 kg preterm neonate following previously reported methods [35]. The airway tree was limited to five bifurcating airway generations (i.e. the trachea corresponds to generation 0). A nylon luer connector (see dashed outline in figure 5) was fitted in the tracheal region of the geometry to mimic the use of a real cuffed endotracheal intubation tube (MLSL035-1; Nordson Medical).

### 3.1. Governing equations

We consider the equations governing fluid flow in modelling the ventilation of the neonate. These consist of the continuity equation, ∇⋅u=0, which describes the conservation of mass for an incompressible fluid, and the Navier–Stokes equations, $\partial u/\partial t+(u\cdot \nabla )u=-\rho^{-1}\nabla p+\nu \nabla^{2}u$, which describe the conservation of momentum for a Newtonian fluid, where **u** is the fluid’s velocity field, *t* is time, *p* is pressure and *ν* = *μ*/*ρ* is the kinematic viscosity. Choosing appropriate scaling with **u** = u*/*U*; $\nabla =\nabla^{*}D$; *t* = 2πft*; and *p* = *μUD*^−1^p*, the momentum equations may be non-dimensionalized such that *α*^2^*∂*u*/∂t* + Re$(u^{*}\cdot \nabla^{*})u^{*}=-\nabla^{*}p^{*}+\nabla^{*2}u^{*}$ for a characteristic length *D* (e.g. airway diameter), frequency *f* and mean velocity magnitude *U*. This leads to the role of two dimensionless parameters, namely the Reynolds number Re = *UD*/*ν* and the Womersley number $\alpha =D/2\sqrt{2\pi f/\nu }$. The Reynolds number measures the importance of inertial effects relative to viscous forces and can help predict the onset of turbulence, while the Womersley number measures the relative importance of unsteadiness (i.e. inertial acceleration) due to changes in ventilation frequency. Characteristic values of *α* and Re are reported in table 1.

We note that, when considering the convective–diffusive equations for a scalar transport (e.g. oxygen in air), similar non-dimensionalization yields the Peclet number, $\mathrm{Pe}=UD/D_{O_{2},\mathrm{air}}$, where $D_{O_{2},\mathrm{air}}$ is the diffusion coefficient of oxygen in air. The Peclet number compares the role of convection relative to diffusion, whereby for Pe ≫ 1 diffusion can be safely neglected. Calculating Pe values within the neonatal tree and a typical ventilation velocity, diffusion is anticipated to play a minor role in the region spanning the trachea (generation 0) to approximately the ninth generation or so, though transient reductions in Pe exist near reversal times between inhalation and exhalation, when velocities approach zero. In turn, we confine our treatment of flows in the model upper airway to the role of convection alone.

### 3.2. Experimental set-up

For our experimental investigation, the CAD model was three-dimensional printed in house via stereo-lithography (Form 2; Formlabs) with a 25 μm layer thickness resolution and sprayed with clear lacquer to form a hydrodynamically smooth wall surface. The print was then cast into a clear silicone resin (Sylgard 184; Corning) and dissolved in an acetone solution (approx. 72 h), resulting in a transparent phantom with a 5 mm diameter inlet at the trachea and 16 1.25 mm outlets at generation 4, as depicted in electronic supplementary material, figure S1. The end of the nylon tube connector (inner diameter 3.94 mm) is fixed 15 mm above the first bifurcation, blocking any backflow in the trachea.

To match the refractive index of the silicone (*n* = 1.4095) and achieve optical transparency, a 42/58 (mass ratio) water/glycerol mixture of density *ρ* = 1150 kg m^−3^ and dynamic viscosity *μ* = 9.66 × 10^−3^ kg/m-s was selected as the working fluid [35]. A linear motor (PS01-23 × 160H-HP-R; Linmot, Switzerland) was used to drive flow in and out of the model via a 3 ml syringe connected to the intubation tube by silicone tubing. Ventilatory scenarios spanning HFV (table 1) were reached by altering the frequency and tidal volume of the prescribed oscillatory flow profile. The frequency and tidal volume product *f* × *V*_*T*_, which is associated with the effectiveness of HFV and CO_2_ removal [53], was kept constant [35], thereby imposing a peak Re constant across all cases investigated. The corresponding peak Reynolds number Re_0_ = 4*V*_*T*_*f*/*D*_0_*ν* (at peak velocity in the trachea) and Womersley number $\alpha =D_{0}/2\sqrt{2\pi f/\nu }$ are also reported in table 1. The resulting flow rates as measured in the trachea are shown in figure 5*b* as a function of time, normalized by the breathing cycle period *T*. Values were taken at a transverse cross-section along the trachea, located approximately 5 mm downstream from the opening of the intubation tube. At the start of the inhalation phase, fluid was injected into the airway at an increasing flow rate until a peak was reached at approximately *t*/*T* = 0.2. During the expiratory phase, the direction of flow reverses, most prominently characterized by the merging of flows emanating from the lower branches back upstream. Owing to the complexity of the geometrical model including variation in distances between the inlet (G0) and each of the individual 16 outlets (G4) of the model, expired flows through daughter branches were asynchronous, resulting in a smearing of the peak expiration time measured in the trachea, as seen in the flattened profile between *t*/*T* = 0.6 and 0.8 in figure 5*b*. The duty cycle, or ratio of inspiratory to expiratory times in a breathing cycle, is approximately 1 : 1 with a peak inspiratory flow rate of 10 ml s^−1^. Typical tidal volumes for neonates range between 1 and 3 ml kg^−1^ body weight, representing approximately 50% or less of the anatomical dead space [35]. Note that, following dynamic similarity, the experimental flow parameters (Re and *α*) correspond to clinical ventilation settings in air scaled by 0.5 and $\sqrt{2}$, respectively. We mention here that the oscillatory profile used in this work is only one of several used in clinical practice, and was chosen for its relative simplicity. While it captures well the behaviours of respiratory flow mechanics under continuous ventilatory conditions, it is less appropriate in exploring the effects originating from other waveform profiles known from clinical practice (e.g. discontinuous interruptions). These are anticipated to affect ensuing velocity fields downstream across the conducting airways.

Prior to experimental measurements, the airway phantom was prepared to ensure optical quality. All liquids were introduced in a ‘liquid-to-liquid’ manner; care was taken to prevent the entry of ambient air into the closed system. The substitution of liquids was carried out by infusing one immediately following the other. Note that even trace amounts of air can often produce bubbles and foam, thereby significantly reducing optical access, especially under high-frequency conditions. Preparation of the model began with acetone, which acted to dissolve and remove any particle residue from previous experiments. Next, ethanol (98%) replaced the acetone to serve as a non-reacting buffer and to reduce the surface tension of the silicon inner walls before the introduction of the more viscous 58% glycerol/water solution. Bovine serum albumin (BSA) was added to the glycerol/water (0.01% volume ratio) and left to rest in the model (2 h) before being washed out with the introduction of a pure glycerol/water solution. A thin layer of BSA remaining on the silicon walls was found to delay the adhesion of fluorescent particles, thereby extending the window of time available to perform experiments. Finally, and immediately prior to initiation of an experiment (i.e. image acquisition), a fluorescent particle-seeded glycerol/water solution was introduced into the model and acted as the working fluid for the duration of the experiments (see below).

The instantaneous (time-resolved) three-dimensional, three-component (3D-3C) velocity fields were extracted using high-speed tomoPIV. Four high-speed cameras (Phantom Mini UX100; 1280 × 1024 pixel, 12 bit) were equipped with 100 mm focal length lenses (Zeiss Milvus, Germany) and arranged in an arc configuration as shown in figure 5*c*. Volume illumination was provided by a 70 mJ dual-head neodymium-doped yttrium lithium fluoride (Nd:YLF) laser (DM30-527DH; Photonics Industries, USA), which was triggered in synchrony with the cameras, thereby acquiring phase-locked, double-frame images. The laser beam was introduced through the side of the model and shaped into a thick slab by an optical arrangement consisting of a beam expander and cylindrical lens. Knife-edge filters were applied to the laser beam after the light optics to cut the light intensity beyond the nominal thickness of the measurement volume and reduce the noise in the subsequent reconstructed signal. The flow was seeded with red fluorescent polystyrene particles (PS-FluoRed; microParticles GmbH, Germany). The mean particle diameter *d*_*p*_ = 10 μm and particle density *ρ*_*p*_ = 1050 kg m^−3^ yielded a corresponding particle relaxation time $\tau_{s}=d_{\;p}^{2}\rho_{\;p}/18\mu_{\;f}$ of 0.6 μs. Estimating the viscous time scale $\tau_{\;f}=\nu_{\;f}/U_{\max}^{2}$ based on the maximum mean flow velocity of 0.7 m s^−1^ allowed us to calculate the particle Stokes number, St = *τ*_*s*_/*τ*_*ν*_ = 0.03. Thus, the seeding particles are expected to accurately follow the flow by satisfying St ≪ 1. The time separation between laser pulses (Δ*t*) was 25 μs, yielding maximum particle displacements between 3 and 8 pixels. The imaging frame rate varied between 500 and 1000 fps, yielding a sufficiently high temporal resolution to capture rigorously flow phenomena occurring over one cycle. Raw image and PIV processing were performed with Davis 10 (LaVision GmbH, Germany) and further analysed in Matlab (Mathworks Inc., USA).

The corresponding measurement volume for tomoPIV was located in the *x*–*y* plane perpendicular to the mean streamwise flow in the trachea, as shown in figure 5*c*. Owing to the arced configuration of the four cameras, Scheimpflug conditions must be met in order to focus the image over the entire field of view. The Scheimpflug criterion was achieved by rotating the lens plane relative to the camera image plane in an iterative method described by Raffel *et al.* [69]. Lens apertures (*f*/4–*f*/5.6) were chosen to maximize light collection and ensure adequate depth of focus to cover the entire measurement volume. Camera calibration was performed by translating a three-dimensional calibration plate (058-1; LaVision GmbH) through the measurement volume in place of the phantom model. The calibration plate was cast into a separate silicon resin block of identical outer dimensions and optical specifications as the model following a method analogous to that described by Buchmann *et al.* [70]. The calibration plate was translated between *z* = −3.5 mm and *z* = 3.5 mm at 0.5 mm intervals (i.e. for a total of 15 planes), with an uncertainty of ±5 μm of the translation stage (RB13M; Thorlabs). An additional volume self-calibration procedure [71] was performed on subsequently recorded raw particle images in the airway phantom to remove any residual calibration disparities. This procedure reduces experimental errors by an additional order of magnitude, from less than 0.5 pixel to less than 0.05 pixels.

The main processing steps to analyse the three-dimensional velocity fields from the raw particle image data are briefly as follows: further details on tomoPIV can be found, for example, in Elsinga *et al.* [72] and Westerweel *et al.* [73], and applications for physiological flows are discussed in Buchmann *et al.* [70]. Several pre-processing steps were first performed in Davis 10 (LaVision GmbH) to eliminate background noise and compensate for variations in intensity across images and between cameras. A geometric mask with the outline shape of the model was first applied to each camera to exclude areas outside the particle-seeded measurement volume. Next, a minimum time filter was subtracted from all images to eliminate background noise (i.e. from static fluorescent particles stuck to the walls), though this filter had minimal contribution owing to the effectiveness of the BSA blocking. A sliding minimum convolution filter was applied with a 3 × 3 pixel window to eliminate variations in laser illumination and to subtract minimum intensity values. The images were normalized by the local 200 pixel average to compensate for intensity differences between the first and the second laser pulse and normalized relative to the first camera and exposure. Gaussian smoothing was applied to improve the visual quality of particles followed by sharpening to reduce the increase in particle size caused from Gaussian smoothing. A 10 pixel subtraction filter was applied to reduce background noise to zero counts and finally all image pixel intensity counts were multiplied by a factor of 10 to fully use the 12 bit dynamic range. After pre-processing, voxel-based three-dimensional particle intensity distributions were reconstructed using six iterations on sets of four images using an enhanced algebraic reconstruction technique [74] (fastMART). Next, a multi-pass cross-correlation technique was employed whereby the interrogation volume decreased from an initial size of $96\times 96\times 96\;{\text{voxels}}^{3}$ to a final size of $16\times 16\times 16\;{\text{voxels}}^{3}$ with an adjacent window overlap of 75%. The resulting vector spacing was 0.2 mm in all directions. Phase-locked mean velocity fields were extracted by averaging 30 oscillatory cycles. Data processing was performed on two 12-core processors (Intel Xeon E5-2630v4; 2.2 GHz) with 128 GB RAM memory requiring approximately 24 h for the full analysis (i.e. preprocessing, volumetric reconstruction and cross correlation) of one complete experimental run (approx. 1000 images).

### 3.3. Numerical set-up

Numerical simulations were performed by solving the governing conservation of mass and momentum (i.e. Navier–Stokes) equations using the finite volume method in ANSYS Fluent. Cyclic flow conditions following a sinusoidal velocity profile were applied at the inlet as defined in electronic supplementary material, table S1. A zero pressure condition was applied at each of the 16 distal outlets of the upper airway so as to closely mimic the experimental boundary conditions. The airway model including the intubation tube was meshed with polyhedral cells and wall prism layers using ANSYS ICEM. Rigorous mesh convergence tests were first performed to select the optimal numerical set-up. Polyhedral meshes ranging between 0.33M and 2.9M cells were investigated. In particular, we independently examined the near-wall refinements as well as refinements of large airway branches, small branches and high-curvature regions. For each of these meshes, mass conservation was verified and velocity values converged to within 0.005% tolerance. Following convergence tests, the chosen mesh consisted of 0.87M polyhedral cells with three near-wall prism layers. Calculations were performed using a coupled pressure–velocity scheme along with a least-squares-based scheme for gradients and a second-order upwind scheme for both velocity and pressure terms. The time step chosen for each scenario was *T*/500 (e.g. 0.2 ms for the *α* = 6.8 case) with peak errors less than 0.7% compared with a time step twice as small (i.e. *T*/1000). Using identical glycerol/water mixture fluid properties (i.e. density and viscosity), we assume the flow to be incompressible, Newtonian and isothermal. The numerical model assumes rigid, stationary walls, thereby neglecting the compliance of the silicone phantom, which fluctuates ±3% in tracheal airway diameter. Furthermore, the outlet conditions in the numerical solution neglect the hydrostatic pressure drop owing to tubing extending beyond the airway outlets of the experimental model, though this discrepancy was found to be negligible considering the much higher typical pressure gradients due to geometric constraints. Further details on our *in silico* methods can be found in a recent study [35].

One of the advantages afforded by CFD is the high-resolution data acquired in the vicinity of the model walls which are suitable for studying the effects of WSS. WSS is defined as ****τ**** = *μ∂***u**/*∂n*|_wall_, where *n* is the normal vector to the inner wall. Since the working fluid in our experimental and numerical models is a viscous water/glycerol mixture, dynamic similarity must be taken into account in order to relate WSS measurements in our model to the clinical setting in air. We non-dimensionalize the WSS, yielding τ* = *μ*(*UD*)^−1^ *∂*u*/∂n* = (*μUD*)^2^*ρ*^−1^ Re *∂*u*/∂n*|_wall_. WSS values measured in water/glycerol correspond to values in air scaled by approximately 2.

## Supplementary Material

Supplementary Information

## Supplementary Material

Movie S1

## Data accessibility

This article has no additional data.
