# Supplementary material for: Ventilation-induced jet suggests biotrauma in reconstructed airways of the intubated neonate
Source: J R Soc Interface. 2020 Jan 8;17(162):20190516. doi: 10.1098/rsif.2019.0516 (PMC7014802; doi:10.1098/rsif.2019.0516)
Supplement: Supplementary Information [file rsif20190516supp1.pdf]

Ventilation-induced jet suggests biotrauma in reconstructed airways of the intubated neonate

Eliram Nof[1], Metar Heller-Algazi[1], Filippo Coletti[2], Dan Waisman[3,4], and Josué Sznitman[1,*]

[1]Department of Biomedical Engineering, Technion â“ Israel Institute of Technology, Haifa 3200003, Israel
[2]Department of Aerospace Engineering and Mechanics, University of Minnesota, Minneapolis, Minnesota 55455, USA
[3]Department of Neonatology, Carmel Medical Center, Haifa 3436212, Israel
[4]Faculty of Medicine, Technion â“ Israel Institute of Technology, Haifa 3200003, Israel
[*]sznitman@technion.ac.il

ABSTRACT

We investigate respiratory flow phenomena in a reconstructed upper airway model of an intubated neonate undergoing invasive mechanical ventilation, spanning conventional to high-frequency ventilation (HFV) modes. Using high-speed tomographic particle image velocimetry, we resolve transient, three-dimensional flow fields and observe a persistent jet flow exiting the endotracheal tube whose strength is directly modulated according to the ventilation protocol. We identify this synthetic jet as the dominating signature of convective flow under intubated ventilation. Concurrently, our *in silico* wall shear stress analysis reveals a hitherto overlooked source of ventilator-induced lung injury as a result of jet impingement on the tracheal carina, suggesting damage to the bronchial epithelium; this type of injury is known as biotrauma. We find HFV advantageous in mitigating the intensity of such impingement, which may contribute to its role as a lung protective method. Our findings may encourage the adoption of less invasive ventilation procedures currently used in neonatal intensive care units.

Keywords: neonates, lung injury, biological fluid mechanics, biotrauma, respiratory distress syndrome

Supplementary Information

Movie S1. Entire duration of inhalation phase in reconstructed upper airways of an intubated preterm neonate undergoing invasive ventilation. 3D velocity vector fields are experimentally extracted via tomographic particle image velocimetry (tomoPIV) and averaged over 30 phase-locked ventilation cycles. Elongated, jet-like regions of high momentum introduced by the intubation tube into the trachea are visualized by plotting iso-surfaces of the normalized velocity magnitude $|\mathbf{u}^*|$. The jets are depicted according to three levels of iso-surfaces: a high velocity core (80% of maximum velocity) sheathed by partially transparent lower velocities, i.e. 60% and 40%, respectively. Three ventilation modes spanning clinically relevant values of HFV (see Table 1 in the main text) are evaluated: α=3.1 (left), α=4.8 (middle) and α=6.8 (right), where α is the dimensionless frequency (Womersley number).

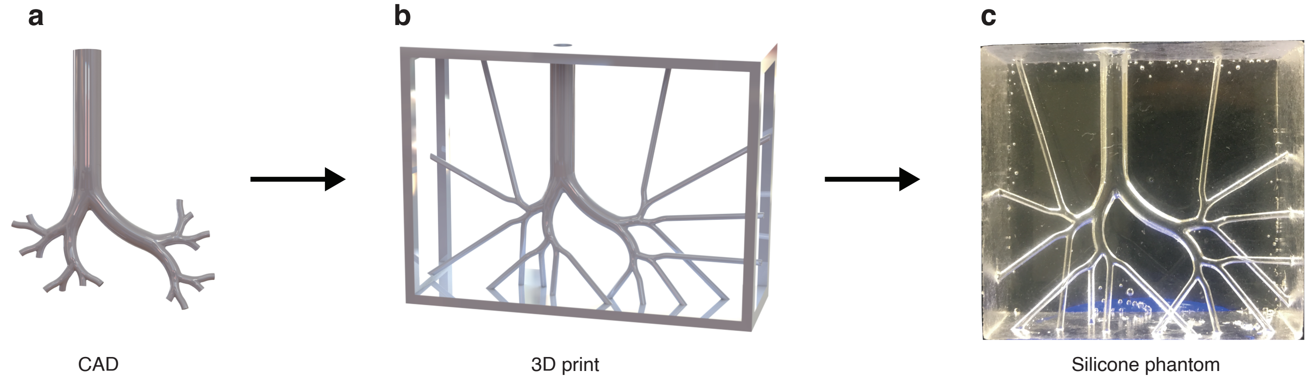

Figure S1. Fabrication process of the reconstructed neonatal upper airway model. (a) A 3D computer aided design (CAD) geometry is printed with a bounding box and extensions (b) into which liquid silicone is poured. (c) Dissolving the positive mold (3D print) results in a transparent phantom.

Table S1. Numerical flow parameters spanning clinically relevant modes of CMV and HFV. Ventilation frequency is given both in dimensionless form (i.e Womersley number, α) and the equivalent ventilation frequency in air, f, calculated via dynamic similarity. Re_0 denotes the mean flow Reynolds number in the trachea (also referred to as airway generation Generation 0) which is kept constant via the ventilation efficiency parameter $f \times V_T$ (frequency $\times$ tidal volume).

f (Hz)	V_T (ml)	α	Re_0
1	10	1.5	300
1.9	5	2.2	300
3.8	2.5	3.1	300
6.3	1.5	3.9	300
9.5	1	4.8	300
12.7	0.75	5.6	300
19	0.5	6.8	300

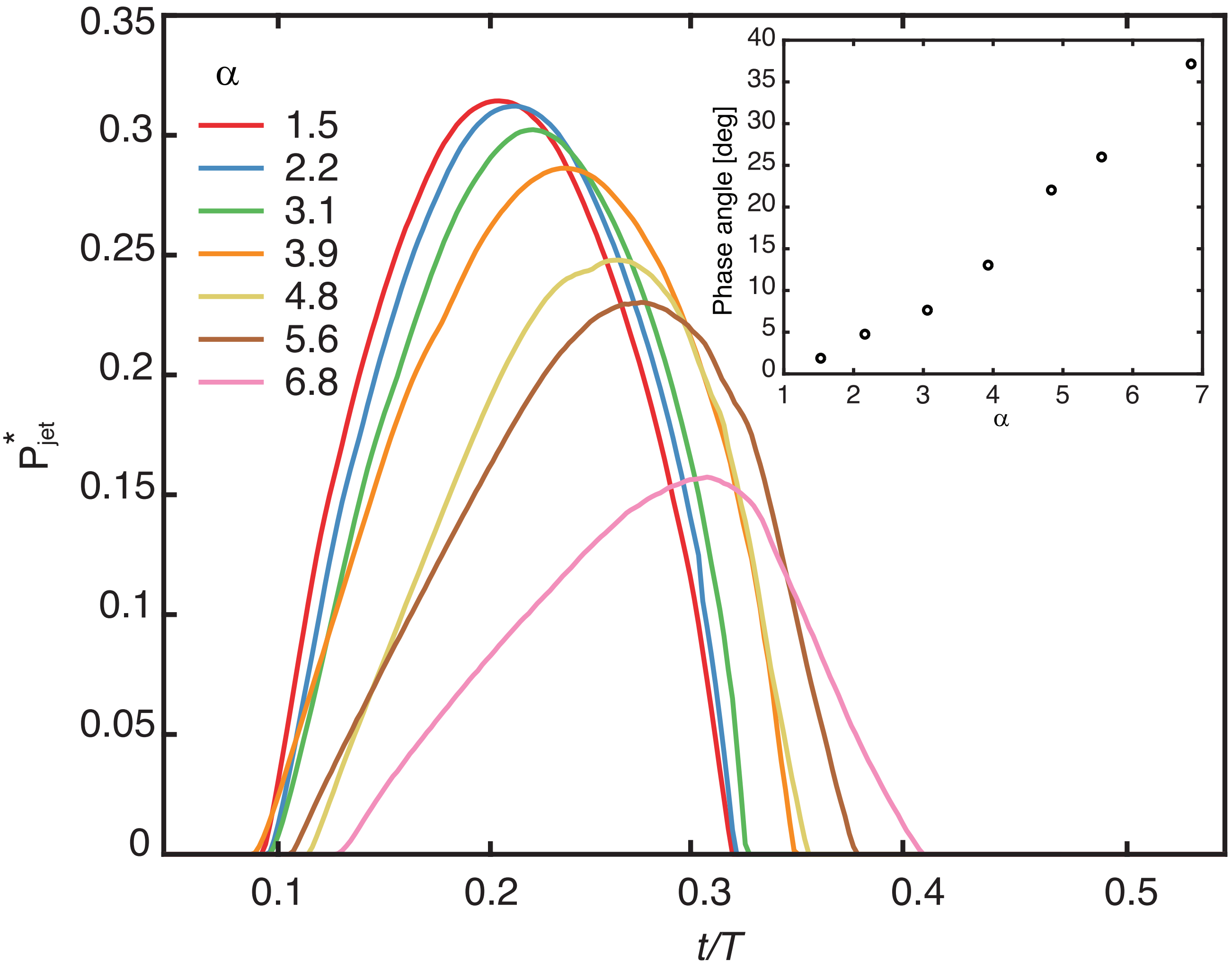

Figure S2. Quantitative momentum analysis on the intubation jet in the trachea from numerical simulations (CFD). Dimensionless P^*_{jet} is plotted over time, t/T, calculated as the momentum within the jet (defined by a 50% maximum velocity magnitude threshold) normalized by the total momentum in the geometry. A decrease in the jet's strength with increasing ventilation frequency (α) is observed and the phase-lag is extracted by plotting the phase angles of the peak P^*_{jet} for each ventilation frequency (see inset). The temporal shift indicates a strong correlation between the jet phenomenon and flow unsteadiness.

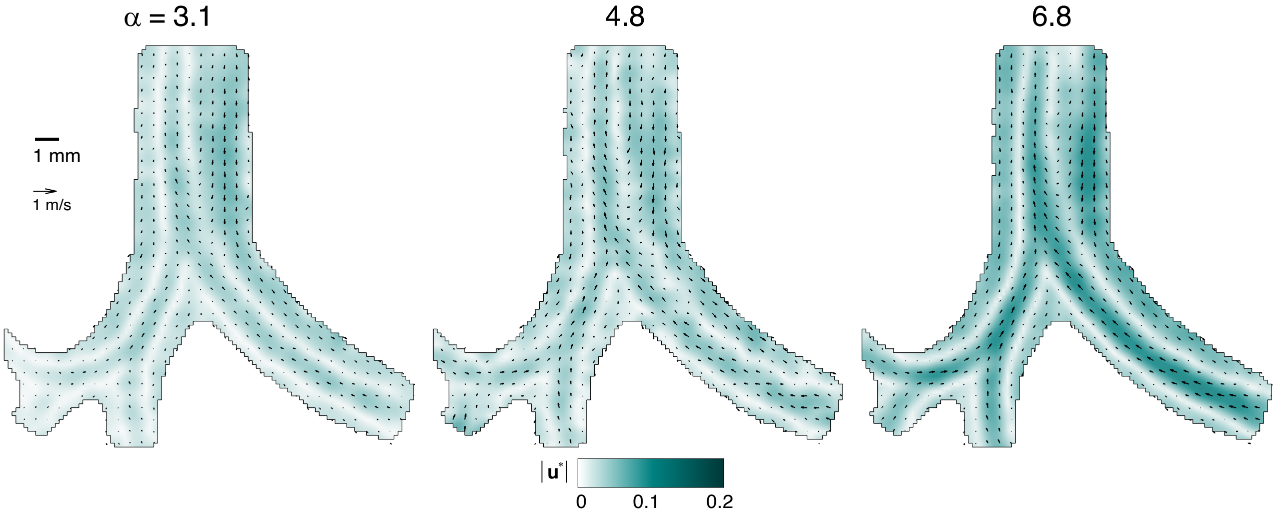

Figure S3. Contour map of the experimentally (tomoPIV) measured velocity magnitudes during the transition from exhalation to inhalation for increasing α values.

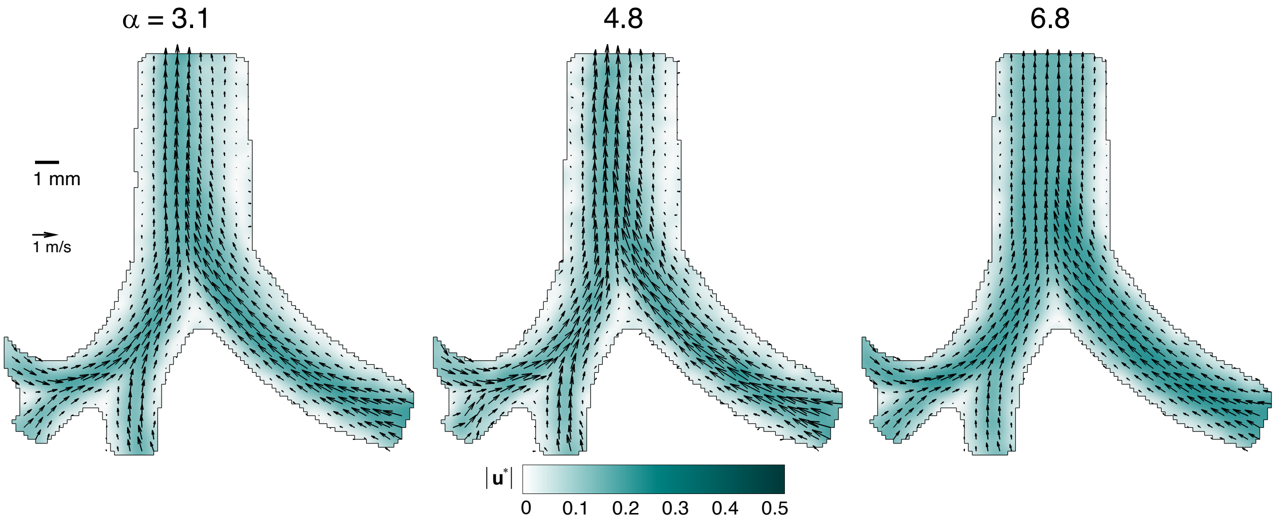

Figure S4. Contour map of the experimentally (tomoPIV) measured velocity magnitudes during peak exhalation for increasing α values.
